# GPR3‐biased signaling is involved in microglial activation in a preclinical Alzheimer’s disease mouse model

**DOI:** 10.1002/alz.095510

**Published:** 2025-01-09

**Authors:** Diego Rolando Hernandez Espinosa, Thais Rafael Guimaraes, Yunhong Huang, Amantha Thathiah

**Affiliations:** ^1^ University of Pittsburgh, Pittsburgh, PA USA; ^2^ APRINOIA Therapeutics, Boston, MA USA

## Abstract

**Background:**

Microglia play a crucial role in clearing amyloid‐beta (Aβ) plaques, one of the primary pathological hallmarks of AD. We previously showed that G protein‐biased signaling by the G protein‐coupled receptor GPR3 reduces soluble Aβ levels and leads to an increase in Aβ plaque compaction and a reduction in Aβ plaque area in the preclinical *App^NL‐G‐F^* AD mouse model. These results suggest a protective microglial response that may limit Aβ plaque formation in G protein‐biased GPR3 AD mice. However, the precise mechanism by which biased GPR3 signaling in microglia restricts the growth of Aβ plaques remains unknown.

**Method:**

We utilized a CRISPR/Cas9 genome editing strategy to mutate six serine/threonine residues in the GPR3 C‐terminus to phosphorylation‐deficient alanine residues to generate a G protein‐biased GPR3 mouse model. We also introduced a hemagglutinin (HA) tag in the N‐terminus of GPR3 to determine the expression and localization of the GPR3 protein *in vivo*. We crossed the *App^NL‐G‐F^* AD mouse model with our GPR3*^HA/HA^* and G protein‐biased GPR3*^HA/Ala^* mice. We then performed bulk RNAseq on cortical brain samples from *App^NL‐G‐F^*, GPR3HA/HA (AD KI), and AppNL‐G‐F, G protein‐biased GPR3 (*Biased AD KI*) mice. To characterize the microglia phenotype, we are conducting Flow cytometry (FC) of *Biased AD KI* relative to *AD KI* brains at 6, 12, and 18 months of age (early‐, mid‐, and late‐stage pathology).

**Result:**

The bulk RNAseq data reveal that microglial activation in *Biased AD KI* mice is consistent with a disease‐associated microglia (DAM) profile. The DAM profile includes upregulation of phagocytosis‐ and endolysosomal‐related genes, such as *Trem2*, *Lyz2*, *Itgax, Cst7*, *Clec7a*, and *Cd68*. Specifically, we observe an upregulation of *Trem2* and *Tyrobp* (DAP12), congruent with our observed increase in the total area occupied by microglia, Aβ plaque coverage area, and Aβ plaque compaction in *Biased AD KI* mice. Preliminary FC analysis also indicates differential expression of DAM markers in microglia isolated from our *Biased AD KI* mice.

**Conclusion:**

Our findings provide compelling evidence for the involvement of biased GPR3 signaling in regulating microglial function and the response to the accumulation of Aβ pathology in AD.

